# Effects of China’s New Rural Cooperative Medical Scheme on reducing medical impoverishment in rural Yanbian: An alternative approach

**DOI:** 10.1186/s12913-016-1660-7

**Published:** 2016-08-22

**Authors:** Mei Sun, Jay J. Shen, Chengyue Li, Christopher Cochran, Ying Wang, Fei Chen, Pingping Li, Jun Lu, Fengshui Chang, Xiaohong Li, Mo Hao

**Affiliations:** 1 Research Institute of Health Development Strategies, Collaborative Innovation Center of Social Risks Governance in Health, Fudan University, 130 Dong An Road, Shanghai, 200032 China; 2 Department of Health Care Administration and Policy, School of Community Health Sciences, University of Nevada, Las Vegas, 4505 S. Maryland Parkway, Box 453023, Las Vegas, NV 89154-3023 USA; 3 Research Institute of Health Development Strategies, Fudan University, 130 Dong An Road, Shanghai, 200032 China

**Keywords:** New rural cooperative medical scheme, Medical impoverishment, Rural, Policy effects, Poverty, Evaluation approach

## Abstract

**Backgroud:**

This study aimed to measure the poverty head count ratio and poverty gap of rural Yanbian in order to examine whether China’s New Rural Cooperative Medical Scheme has alleviated its medical impoverishment and to compare the results of this alternative approach with those of a World Bank approach.

**Methods:**

This cross-sectional study was based on a stratified random sample survey of 1,987 households and 6,135 individuals conducted in 2008 across eight counties in Yanbian Korean Autonomous Prefecture, Jilin province, China. A new approach was developed to define and identify medical impoverishment. The poverty head count ratio, relative poverty gap, and average poverty gap were used to measure medical impoverishment. Changes in medical impoverishment after the reimbursement under the New Rural Cooperative Medical Scheme were also examined.

**Results:**

The government-run New Rural Cooperative Medical Scheme reduced the number of medically impoverished households by 24.6 %, as well as the relative and average gaps by 37.3 % and 38.9 %, respectively.

**Conclusions:**

China’s New Rural Cooperative Medical Scheme has certain positive but limited effects on alleviating medical impoverishment in rural Yanbian regardless of how medical impoverishment is defined and measured. More governmental and private-sector efforts should therefore be encouraged to further improve the system in terms of financing, operation, and reimbursement policy.

## Background

Illness and impoverishment often go hand-in-hand [1]. For low-income individuals, medical expenses can consume a significant proportion of their income, with any subsequent loss of income due to illness then compounding the financial burden [2]. This vicious cycle may continue, since illness and the related medical expenses can force an individual into poverty, leading in turn to further illness and even more severe impoverishment. The World Health Organization (WHO), World Bank (WB), United Nations, and other nongovernmental organizations, for example, have strongly advocated and invested heavily in health care to reduce poverty associated with medical care [3–5]. Indeed, providing health insurance coverage for the rural population is increasingly regarded as not only an important health improvement measure but also an important impoverishment reduction strategy [6].

In the 1970s, China successfully created a community-based rural cooperative medical scheme covering as much as 90 % of all villages by the middle part of the decade, including widespread financial mechanisms for farmers in rural China to access basic health services [7]. However, with the implementation of the Household Responsibility System and the transition from a planned to a market economy, this scheme collapsed by the end of the decade, leaving 90 % of rural residents without health insurance coverage. During the 1980s and 1990s, efforts were made to restore the cooperative medical care system by financing through town-level budgets, but progress was slow [8, 9]. By 2003, nearly 80 % of rural residents were still not covered by any health insurance program [10], meaning that medical care was a heavy economic burden for farmers and medical impoverishment remained a growing problem in rural China.

The 2003 National Health Survey [11] found that almost half (46 %) of rural residents who were ill did not seek health care. Among these individuals, 40 % cited cost as the main reason, while 22 % of those being advised hospitalization refused to be admitted because they could not afford treatment. Moreover, about 35 % of those hospitalized discharged themselves against their doctors’ advice because of high hospitalization expenses. Indeed, some researchers estimated that medical expenses accounted for 30–40 % of poverty [11, 12].

In 2003, the Chinese government launched the New Rural Cooperative Medical Scheme (NRCMS), a government-run system that differed from the previous one in two aspects: (i) farmers contributed to a county-level rather than a town-level financing pool [13]; and (ii) participation was voluntary rather than mandatory. One of the main goals of the system is to address catastrophic medical payments [14]. Since the launch of the NRCMS, access to and utilization of medical care among rural residents has improved [8, 15]. Some studies have evaluated the degree to which the NRCMS alleviates medical impoverishment [9, 16] by using the definition and measurement developed by the WB. However, given that defining and measuring medical impoverishment precisely is the most critical step in studying the problem, this study developed an alternative approach, especially for the NRCMS. It evaluated the degree to which the NRCMS alleviated the level of poverty by particularly taking into account the target of catastrophic medical care set by the new system. The results were then compared with those of the WB approach, using the 2008 data of Yanbian, Jilin province.

## Methods

### The World Bank approach

**Fig. 1:**
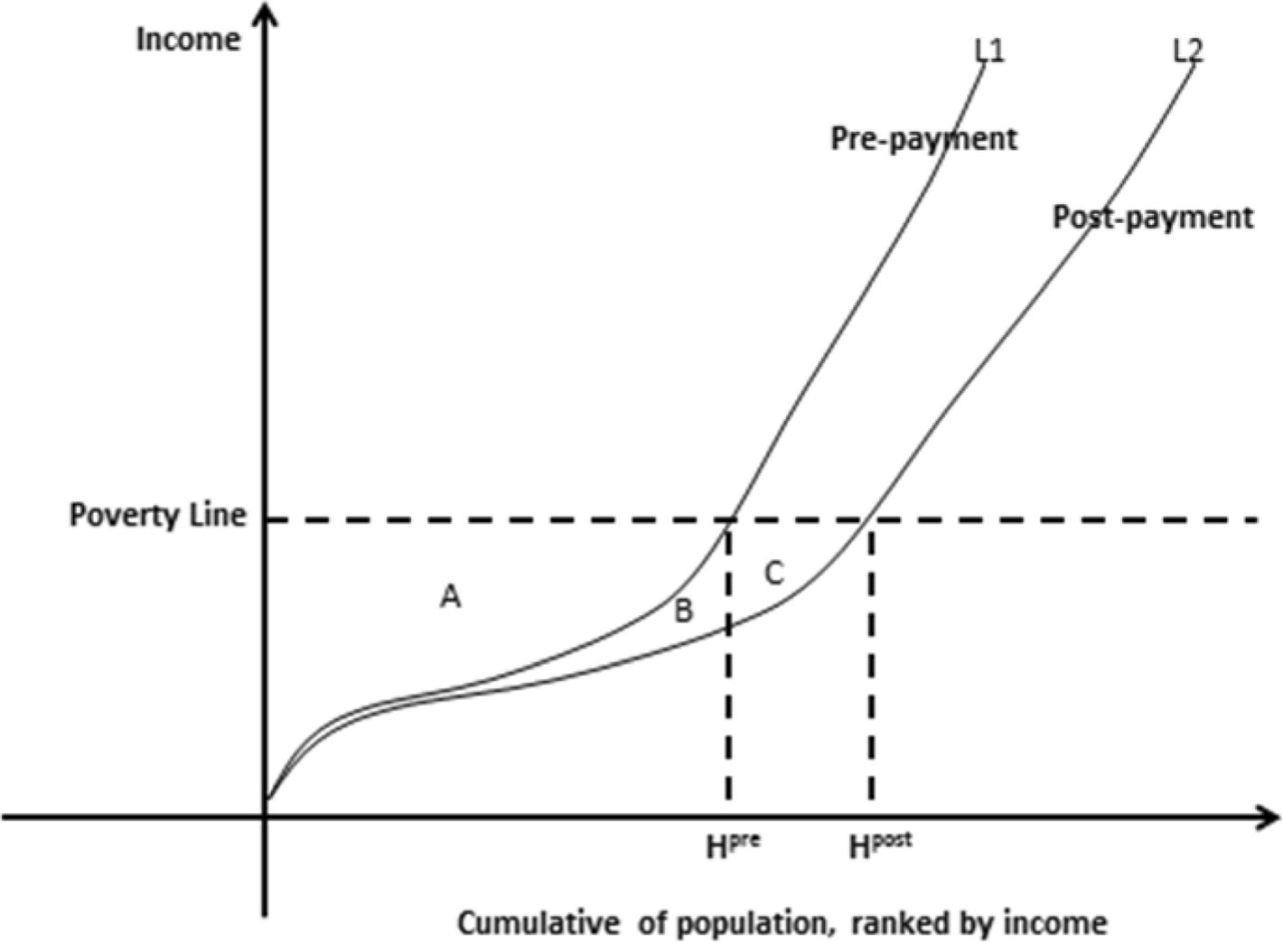
The WB approach

The WB approach to health-care payments and adjusted poverty measures is shown in Fig. 1 [17]. Curve L1 reflects the poverty status of specific populations before paying health-care expenses. Hpre (poverty head count) shows the number of poor people before such payments being made. Curve L2 reflects the poverty status of the population after paying medical expenses. Hpost represents the number of poor people after such payments is made. Therefore, Hpost – Hpre represents the increase in the number of poor people after being subject to medical expenses, or “medical impoverishment” as it is termed herein. G_a_, the area of A, represents the poverty gap without out-of-pocket (OOP) medical expenses. Gb is the gap contribution of OOP medical expenses to those already defined poor. Gc is the contribution to the poverty gap of the newly impoverished due to OOP medical expenses.

In summary, under the WB approach, an income originally above the poverty line could drop below the poverty line after the payment of medical expenses.

### Our definitions and measures

By using the WB approach to evaluate the degree to which the NRCMS alleviates medical impoverishment, we find two kinds of individuals in regard to the change in their impoverishment statuses after encountering medical payment. For example, assume that the poverty line is at 5,000 Yuan per annum, while person A’s income is 4,000 Yuan and his/her medical expenses are 10,000 Yuan. Therefore, A belongs to the poverty population, as his/her income is already below the poverty line. That is, his/her medical expenses do not change him/her from non-poor to poor or vice versa. According to the NRCMS principle (i.e., sharing the cost of catastrophic illness), A is a typical person the NRCMS targets. Second, assume person B’s income is 5,010 Yuan, which becomes 4,990 Yuan after the payment of medical expenses of 20 Yuan. Based on the WB approach, B does not belong to the poverty population before payment, but becomes poor after the payment of medical expenses (i.e., B is driven into medical impoverishment). However, as one of the key features of the NRCMS is to cover the costs of catastrophic medical care as opposed to day-to-day costs, B may not be among the NRCMS’s target population. The WB approach thus calculates the change in poverty status after the payment of medical expenses from *an economics perspective*, but it is not specific enough to evaluate the effects of the NRCMS.

On the other hand, the NRCMS focuses on the prevention of medical impoverishment associated with costs of catastrophic medical care. Therefore, rural residents’ health-care expenses beyond a certain level of their income need to be considered. The target population of the NRCMS should include not only those who fall below the poverty line after incurring medical expenses but also those below the poverty line who would then fall deeper into poverty. To measure medical impoverishment, we assume that the household is the basic economic unit that contracts with the government for farming and other economic activities in rural China. Medical impoverishment is thus defined as a household’s inability to pay OOP medical expenses above a certain level of its discretionary income.

A number of absolute and relative measures are used to define the poverty line. According to the goals of NRCMS, we use 50 % of the per capita income of rural residents (I) in the sample area as the individual poverty line (Li) conservatively [18] as the Organization for Economic Cooperation and Development (OECD) used [19]; the household poverty line (Lh) is thus equal to the individual poverty line multiplied by the number of individuals in the household (Nh), as shown below:

1 $$ Li=\frac{1}{2}\times I $$

2 $$ Lh=Nh\times Li $$

Ability to pay (ATP) is introduced to distinguish persons such as A and B, mentioned previously. ATP, from the individual perspective, is defined as the amount remaining after nondiscretionary consumption has been subtracted from annual per capita income [20]. Two assumptions usually hold in China: (1) almost everyone can pay the entire amount remaining after nondiscretionary consumption for medical expenses; and (2) the household is always the basic economic unit in rural China, not only for contracting with the government but also when someone in the household incurs medical bills. Therefore, we choose to use the ATP of a household, which is the individual’s ATP multiplied by the number of persons in the household, as shown below:

3 $$ \mathrm{R}=\mathrm{N}\mathrm{h}\times \left(\mathrm{I}-\mathrm{L}\mathrm{i}\right)=\mathrm{N}\mathrm{h}\times \mathrm{I}-\mathrm{L}\mathrm{h} $$

where R represents average household ATP, Li represents the individual poverty line, I represents per capita income, Nh indicates the number of persons in a household, and Lh indicates the household poverty line.

Three measures are used to assess the level of medical impoverishment. First, the rate of medical impoverishment, reflecting the overall level of poverty among a population, is defined as

4 $$ U=\frac{P}{N} $$

where U indicates the poverty headcount ratio, P represents the number of households below the household poverty line (i.e., households with medical expenses [Mi] greater than the maximum annual average household ATP [R] and an income level below the poverty line after paying medical bills [Lh]), and N indicates the number of households in the population.

The second is the relative poverty gap, which is used to reflect the depth of poverty among the poverty sub-population. This is defined as the ratio of total medical care expenses exceeding total ATP to total ATP for medically impoverished households. The specific formula is as follows:

5 $$ S=\frac{{\displaystyle {\sum}_{i=1}^P\left(Mi-Ri\right)}}{{\displaystyle {\sum}_{i=1}^PRi}} $$

where **S** is the relative poverty gap, **Mi** represents the i-th household’s OOP medical expenses, **Ri** indicates the i-th household’s ATP, and **P** indicates the number of medically impoverished households.

Third, we calculate the average poverty gap to measure the overall poverty scope and depth among the entire population. This is defined as the ratio of the total ATP of households in poverty to the total ATP of the entire population. The specific formula is as follows:

6 $$ S\hbox{'}=\frac{{\displaystyle {\sum}_{i=1}^P\left(Mi-Ri\right)}}{{\displaystyle {\sum}_{i=1}^NRi}} $$

where **S’** is the average poverty gap, **Mi** represents the i-th household’s OOP medical expenses, **Ri** indicates the i-th household’s ATP, and **N** is the number of households in the entire population.

Finally, we use two indicators to measure the degree to which the NRCMS alleviates medical impoverishment. The first is the change in the poverty headcount ratio, which is calculated as

7 $$ \Delta \mathrm{U}={U}_{pre\_ reimbursement-}{U}_{post\_ reimbursement} $$

8 $$ \Delta \mathrm{U}\%=\frac{\Delta U}{U_{pre\_ reimbursement}}\times 100\% $$

In this calculation, U_pre_reimbursement_ is the poverty headcount ratio before receiving NRCMS reimbursement and U_post_reimbursement_ represents the poverty headcount ratio after the reimbursement, while Δ*U* % is the percentage change in the poverty headcount ratio after the reimbursement.

The second indicator is the difference between the poverty gaps before and after NRCMS reimbursement:

9 $$ \Delta \mathrm{S}=\frac{{\displaystyle {\sum}_{i=1}^P\left(M{i}_{\mathrm{pre}\_ reimbursement}-Ri\right)}}{{\displaystyle {\sum}_{i=1}^PRi}}-\frac{{\displaystyle {\sum}_{i=1}^P\left(M{i}_{post\_ reimbursement}-Ri\right)}}{{\displaystyle {\sum}_{i=1}^PRi}} $$

10 $$ \Delta {\mathrm{S}}^{\prime }=\frac{{\displaystyle {\sum}_{i=1}^P\left(M{i}_{pre\_ reimbursement}-Ri\right)}}{{\displaystyle {\sum}_{i=1}^NRi}}-\frac{{\displaystyle {\sum}_{i=1}^P\left(M{i}_{post\_ reimbursement}-Ri\right)}}{{\displaystyle {\sum}_{i=1}^NRi}} $$

11 $$ \Delta \mathrm{S}\%=\Delta \mathrm{S}\div \left[\frac{{\displaystyle {\sum}_{i=1}^P\left(M{i}_{\mathrm{pre}\_ reimbursement}-Ri\right)}}{{\displaystyle {\sum}_{i=1}^PRi}}\right]\times 100\% $$

12 $$ \Delta {\mathrm{S}}^{\prime}\%=\Delta {\mathrm{S}}^{\prime}\div \left[\frac{{\displaystyle {\sum}_{i=1}^P\left(M{i}_{\mathrm{pre}\_ reimbursement}-Ri\right)}}{{\displaystyle {\sum}_{i=1}^NRi}}\right]\times 100\% $$

In these calculations, Mi_pre_reimbursement_ represents OOP medical expenses before NRCMS reimbursement; Mi_post_reimbursement_ represents OOP medical expenses after the reimbursement; and Δ*S* ' represent the absolute differences in the relative and average poverty gaps, respectively, between the pre- and post-reimbursement levels; and Δ*S* % and Δ*S* ' % represent changes in the percentages of the relative and average poverty gaps, respectively, between the pre- and post- reimbursement levels.

### Case study

We used a cross-sectional study to illustrate our approach and compare the results with those of the WB method. Household health-care utilization and expenditure surveys were conducted in January 2008 in eight counties in Yanbian Korean Autonomous Prefecture, Jilin province, China. The surveys gathered individual and household demographics, socioeconomic characteristics, two-week morbidity, six-month chronic illness prevalence, health service needs and utilization, and health-care expenses [21].

We used the probability proportionate to size sampling method to randomly select villages in each of the eight counties. Around 10 % of the villages in each county were selected. In each village, we first selected a random household from a list of household register numbers. The next household was the one that was closest to the first household in terms of walking steps. If more than one household was equally close to the first household, the one on the left (for a person stepping out of the house) was selected. The process continued until a sufficient number of households had been surveyed. About 10 % of the households in a village were selected. A total of 6,135 individuals in 1,987 households were surveyed.

Further, we used Myer’s Index to test the representative character of our survey data in terms of age structure based on China’s 2000 Census data [22]. We obtained a Myer’s Index of 3.38, which showed that our data represented the age distribution of national population well. A Myer’s Index greater than 60 indicates significant differences in age structures between one sample and another.

This study was approved (IRB#08–03–0130) by the Medical Research Ethics Committee, School of Public Health, Fudan University (IRB00002408&FWA00002399). A verbal consent was obtained from all the respondents before conducting the interviews.

## Results

Table 1 displays the unadjusted socio-demographics and health-care utilization of farmers. The average annual household income was 22,392 Yuan (1CNY = 0.944USD in 2008), while 9.5 % of households were below the poverty line of 6,141 Yuan per annum (around $2.42 per day). Altogether, 96.8 % of households participated in the NRCMS, while the rates of two-week and annual outpatient visits were 18.4 and 94.5 %, respectively, and the annual hospitalization rate was 4.7 %. Overall, the NRCMS reimbursed 11.2 % of total medical expenses, 3.2 % of outpatient expenses, and 40.2 % of inpatient expenses.

**Table 1:** Socio-demographics and Health-Care Characteristics of Households and Individuals

Variable	Amount
Households, total count	1,987
Individuals, total count	6,135
Socio-demographics	
Age group	
0–20	17.4 %
21–44	37.9 %
45–64	35.3 %
≥65	9.4 %
Sex	
Female	48.4 %
Male	51.6 %
Elementary school education or less	35.2 %
Annual household income, mean, Yuan	22,392
Poverty line	6,141 Yuan (around $2.42/day)
Households below the poverty line	9.5 %
Health care, reimbursement, and expenses	
Households participating in NRCMS	96.8 %
Two-week outpatient visit	18.4 %
Annual outpatient visit	94.5 %
Hospitalization	4.7 %
Annual reimbursement	11.2 %
Annual outpatient reimbursement percent	3.2 %
Annual inpatient reimbursement	40.2 %

Table 2 shows changes in household income and OOP medical expenses for medically impoverished households after NRCMS reimbursement. In total, 134 households were considered in medical impoverishment before NRCMS reimbursements, and the number reduced to 101 after reimbursements. Of the 134 households, average household income was 10,103 Yuan and average OOP medical expenses were 10,711 Yuan. Of the 101 households, average household income was 9,376 Yuan and average OOP medical expenses were 9,918 Yuan.

**Table 2:** Changes in Income and Medical Impoverishment after NRCMS Reimbursement

	Before reimbursement	After reimbursement
Number of medically impoverished households	134	101
Average persons per household	2.6	2.7
Average household income, Yuan	10,103	9,376
Per capita income, Yuan	3,798	3,511
Average medical expenses, Yuan	10,711	9,918

Table 3 presents a comparison of the degree to which the NRCMS has alleviated medical impoverishment based on our approach and the WB method. According to our approach, the NRCMS reduced the number of medically impoverished households by 24.6 % and the relative and average gaps by 37.3 and 38.9 %, respectively. According to the WB approach, the NRCMS reduced the number of medically impoverished households by 13.4 % and the relative and average gaps by 20.6 and 20.3 %, respectively.

**Table 3:** Effects of the NRCMS on Medical Impoverishment

Indicator	Before reimbursement	After reimbursement	Difference	Percentage change
New method				
Number of households in poverty	134	101	–33	–24.6 %
Percentage of households in poverty, %	6.7	5.1	–1.6	–23.9 %
Relative gap	82.0	51.4	–30.6	–37.3 %
Average gap	5.4	3.3	–2.1	–38.9 %
WB approach				
Number of households in poverty	253	219	–34	–13.4 %
Percentage of households in poverty, %	12.7	11.0	–1.7	–13.4 %
Relative gap	54.5	43.3	–11.2	–20.6 %
Average gap	6.9	5.5	–1.4	–20.3 %

Table 4 shows results of households identified by WB’s but not our approach. Before NRCMS reimbursement, the WB approach did not identify 27 households who were already poor and whose medical expenses were more than the household ATP; but misidentified 146 households whose medical expenses were less than their ATP. After NRCMS reimbursement, the WB approach did not identify 22 households in medical impoverishment and misidentified 140 medically impoverished households as well.

**Table 4:** Medically Impoverished Households Identified by the WB Approach but not by the New Approach

	Before reimbursement		After reimbursement	
	Medical Expenses > ATP	Medical expenses < ATP	Medical expenses > ATP	Medical expenses < ATP
Number of households	27	146	22	140
Average number of residents in household	2.6	3.1	2.6	3.0
Average household income, Yuan	4,201	8,721	4,247	8,480
Average OOP medical expenses, Yuan	9,003	3,418	8,530	3,276

## Discussion

The findings of this study indicate that the New Rural Cooperative Medical Scheme in rural Yanbian has alleviated medical impoverishment in 25 to 38 % of the households in the sampled area, indicating that the status of most households in poverty remains unchanged, which is similar to the findings of other research studies [6, 21, 23]. There are several explanations for the limited effect of the NRCMS. First, NRCMS reimbursement has been unable to keep up with the rapid growth in health-care expenses. China’s health-care expenditure, in recent years, has escalated at 16 % annually, 7 % faster than GDP growth; and patients’ out-of-pocket health expenses have grown at an average annual rate of 15.7 % [24, 25]. Although the funding level has risen considerably, from 40 Yuan to 120 Yuan annually [26], the county-based NRCMS, given their lack of experience in risk estimation and projection, in general, mainly aims to break even financially, which results in limited benefit coverage, high deductibles, and a low cap for reimbursement [27].

Second, the NRCMS focuses on covering catastrophic medical events, most of which involve hospitalization. Payments for outpatient care are largely neglected in its reimbursement policies [28]. As the prevalence of chronic diseases has gone up in recent years, the volume of outpatient visits has also increased [29]. Although charges per outpatient visit are relatively low compared with inpatient care charges, cumulative medical bills from outpatient visits can become a significant financial burden on households, too [9]. In some cases, annual cumulative outpatient expenses may even be higher than inpatient expenses [30].

Both the World Bank method and our alternative approach suggest that the effects of the NRCMS are similar. The major difference between the two approaches is derived from the definition of medical impoverishment. The WB approach focuses on the status of total income and, therefore, is generally applicable for measuring poverty. Our approach defines medical impoverishment based on an individual’s ability to pay for the medical expenses. As a consequence, our approach seems to more accurately evaluate the effect of NRCMS in rural China than does the WB approach. The WB approach identifies poor households whose original incomes are slightly above the poverty line before incurring medical care but then fall slightly below the poverty line after paying moderate medical expenses. Strictly speaking, these households do not suffer “real” impoverishment caused by high medical bills because they are already on the verge of poverty before such expenses. In fact, this proportion of the population requires greater social welfare and other public assistance beyond mere support from the NRCMS. Because the NRCMS was created to reduce the financial risk of high medical expenses due to illness, our ATP-based approach, seems to more accurately capture the “real” impoverishment merely caused by expensive medical bills among rural residents in China.

Moreover, the WB approach seems to miss households whose original household income is below the poverty line and their medical expenses exceed their maximum amount of ability to pay. With the addition of high medical bills, achieving financial security is even more challenging for those who already live in poverty. These vulnerable populations need help the most from insurance programs like the NRCMS. Again, the WB approach focuses on the poverty headcount ratio and poverty gap differences before and after incurring medical expenses. In contrast, our approach combines the WB approaches based on adjusted poverty measures and catastrophic payments for health care.

Although this combination could be particularly useful to evaluate the effects of NRCMS in rural China, whether it is appropriate to measure poverty in other countries merits more empirical research, in consideration of the limitation of relative poverty line [31]. In addition, we collected outpatient care information by asking the famers to recall outpatient visits for the last two weeks as opposed to hospitalization that was based on the last 12 month recall. Although we used the bootstrapping method to generalize the two-week information to annual outpatient care utilization and expenses, variations may still exist. Third, our study was based on one ethnic minority region in China, which may discount the generalizability of the findings.

## Conclusions

In this study, we find that the New Rural Cooperative Medical Scheme has achieved certain but limited impact on alleviating medical impoverishment in rural Yanbian, regardless of how medical impoverishment is defined and measured. Further, the new approach we present seems to be able to capture medical impoverishment more accurately among the target population of the new system. Policies should encourage system improvement in the areas of financing through the combination of government subsidies and rural residents’ per capital annual premium, better benefits coverage and reimbursement schedules.

## Abbreviations

ATP: Ability to Pay
NRCMS: New Rural Cooperative Medical Scheme
OECD: Organization for Economic Cooperation and Development
OOP: Out-of-pocket
WB: World Bank
WHO: World Health Organization
